# Intraoperative hypotension and postoperative delirium in elderly male patients undergoing laryngectomy: a single-center retrospective cohort study

**DOI:** 10.1016/j.bjane.2024.844560

**Published:** 2024-09-12

**Authors:** Yiru Wang, Kaizheng Chen, Min Ye, Xia Shen

**Affiliations:** Fudan University, Eye & ENT Hospital, Department of Anesthesiology, Shanghai, China

**Keywords:** Hypotension, Laryngectomy, Postoperative delirium, Risk factor

## Abstract

**Background:**

Postoperative delirium (POD) is a common, transient postoperative cognitive dysfunction in elderly patients. The relationship between POD and intraoperative hypotension remains unclear. This study aims to determine if intraoperative hypotension predicts POD in elderly male patients undergoing laryngectomy.

**Methods:**

This study included male patients over 65 years old who underwent laryngectomy between April 2018 and January 2022. The Confusion Assessment Method (CAM) was used to diagnose delirium. Intraoperative hypotension was defined as a Mean Arterial Pressure (MAP) during surgery that was less than 30% of the preoperative level for at least 30 minutes. The relationship between intraoperative hypotension and POD incidence was adjusted for patient demographics and surgery-related factors.

**Results:**

Out of 428 male patients, 77 (18.0%) developed POD, and 166 (38.8%) experienced intraoperative hypotension. Surgery duration ≥ 300 minutes (OR = 1.873, 95% CI 1.041–3.241, *p* = 0.036), intraoperative hypotension (OR = 1.739, 95% CI 1.039–2.912, *p* = 0.035), and schooling (OR = 2.655, 95% CI 1.338–5.268) were independent risk factors for POD. The association between intraoperative hypotension and POD was significantly influenced by surgery duration (*p* for interaction = 0.008), with a stronger association in prolonged surgeries (adjusted OR = 4.902; 95% CI 1.816–13.230).

**Conclusions:**

Intraoperative hypotension and low education level are associated with an increased risk of POD in elderly male patients undergoing laryngectomy, especially with prolonged surgery duration.

## Introduction

As the population ages, the number of elderly individuals requiring surgical treatment is increasing.^1^, ^2^, ^3^ Elderly patients often experience more age-related complications due to frailty and physical deterioration.^4^^,^^5^ Postoperative cognitive impairment is a common complication in elderly patients.^6^ Postoperative delirium (POD), characterized by severe fluctuations in cognitive function and attention deficits, is a transient form of this impairment.^7^^,^^8^ POD can lead to adverse outcomes, including prolonged hospital stays, increased costs, postoperative mortality, poor long-term quality of life, and even long-term cognitive impairment.^9^, ^10^, ^11^

The causes of POD are multifactorial. Clinical studies have identified several unmodifiable risk factors, including sensory deficits, impaired functional status, cognitive impairment, comorbidities, and frailty.^7^^,^^12^ Modifiable risk factors, such as medication use, type of anesthesia, and perioperative management strategies, also play a role. Since treatment options for delirium are limited, prevention strategies targeting these modifiable factors are crucial.^13^^,^^14^

Intraoperative hypotension is a common side-effect of general anesthesia and is associated with postoperative adverse events like acute kidney injury and myocardial ischemia.^15^^,^^16^ While the brain can typically respond to ischemia and hypoxia,^17^^,^^18^ this capacity is diminished in elderly patients due to reduced arterial elasticity and other comorbidities.^19^^,^^20^ Prolonged intraoperative hypotension can compromise cerebral perfusion, potentially leading to neurological complications.^21^^,^^22^ While several studies have explored the relationship between intraoperative hypotension and POD, their results have been inconclusive.^2^^,^^23^^,^^24^

Our study uniquely focuses on elderly patients undergoing laryngectomy, a procedure involving significant physiological stress and extended duration, both of which may influence POD incidence.^25^ By examining this specific surgical population, we aim to provide detailed insights into the interaction between intraoperative hypotension and other potential risk factors for POD, such as surgery duration, patient demographics, and dexmedetomidine use.^25^

Therefore, in this retrospective study, we investigated whether intraoperative hypotension would predict the onset of POD in elderly patients undergoing laryngectomy. We also considered other potential risk factors, including education level and preoperative cognitive status, to better understand their roles in the development of POD.

## Methods

### Data source

This retrospective cohort study was conducted at the Eye and Ear Nose Throat (ENT) Hospital, Fudan University. The study was approved by the hospital's review board, and informed consent was waived. The study adhered to the Declaration of Helsinki and followed STROBE guidelines for observational studies. Data were obtained from 812 patients who underwent laryngectomy at the Eye and ENT Hospital from April 2018 to January 2022. Patients’ characteristics and perioperative data were obtained from the hospital information system (appref-ms) and the anesthetic system (DoCare V5.0).

### Study population

The enrollment criteria were as follows: male patients aged 65 years or older who underwent laryngectomy under general anesthesia. Exclusion criteria included neurodegenerative diseases, history of delirium, history of mental illness, and missing data for potential confounding variables. Female patients were excluded due to the very low prevalence of laryngeal cancer in females (1.3%)^26^ and their frequent refusal to participate in the study. This decision was made to ensure a sufficient sample size and homogeneous study population.

### Data collection

Detailed information on patient demographics and clinical characteristics was collected: cancer stage, education level (highest degree completed, with high school corresponding to 12 years of schooling), MMSE scores (Mini-Mental State Examination) (Appendix 1), CIRS scores (Cumulative Illness Rating Scale) (Appendix 2), comorbidities (history of cerebral stroke, hypertension [systolic BP ≥ 140 mmHg or diastolic BP ≥ 90 mmHg], diabetes mellitus), smoking status (regular tobacco use), drinking status (regular alcohol consumption), family history of dementia, preoperative poor sleep quality (self-reported sleep disturbances in the month prior to surgery), surgery type, surgery duration, amount of fluid infusion, and intraoperative dexmedetomidine use. Intraoperative Mean Arterial Pressure (MAP) data was obtained from the anesthetic system (DoCare V5.0), which documented at a 5-min interval.

Consistent with our previous study,^25^ intraoperative hypotension was defined as a MAP decrease of 30% or more below the preoperative baseline, lasting for at least 30 minutes. We redefined intraoperative hypotension as a MAP decrease of 30% or more below the preoperative baseline, lasting for at least 30 minutes. This definition was based on prior studies that have shown significant associations between sustained reductions in MAP and delirium.^27^

Patients with a MMSE score < 27 were considered to have preoperative cognitive impairment.^28^ The score of the CIRS was used to assess preoperative comorbidity, patients with a score ≥ 8 were considered high comorbidity.^8^ Low education level was defined as patients graduated from less than high school (corresponding to 12 years of schooling). Prolonged surgical duration was defined as surgery duration ≥ 300 minutes. The amount of rehydration ≥ 3000 mL was considered to have excessive fluid replacement.

### Outcome

Since symptoms of POD are often worse at night,^29^ patients were interviewed daily using the Confusion Assessment Method (CAM) diagnostic algorithm^30^ (Appendix 3) between 4 p.m. and 6 p.m. during the first five days after surgery. For patients in ICU, the CAM-ICU was used. Accurate detection of delirium with the CAM requires evaluation of four features: acute onset with a fluctuating course, inattention, disorganized thinking, and altered level of consciousness. Patients meeting the CAM criteria on any assessed day were considered positive for delirium. Prior to the initial study, the investigators conducting the postoperative follow-up and delirium assessment had received specialized training related to psychiatry.

### Statistical analysis

Data were analyzed using SPSS 22.0. A *p*-value < 0.05 (two-sided) was considered statistically significant. Normally distributed data were presented as means with Standard Deviations (SD) and analyzed with two-sample Student's *t*-tests. Skewed continuous data were presented as medians with Interquartile Ranges (IQR) and evaluated using Wilcoxon signed rank tests. Categorical variables were expressed as numbers (percentage) and tested using χ^2^ test, Fisher's exact test, or Cochran-Mantel-Haenszel χ^2^ test. Variables associated (*p* < 0.2) with POD in univariate analysis and potential variables (older age, CIR > 8, and no intraoperative dexmedetomidine use) which have been shown as risk factors in previous studies^8^^,^^25^ were used as candidate risk factors for the stepwise logistic regression multivariate models.

We performed a stratified analysis based on the variables associated (*p* < 0.05) with POD in multivariable logistic regression analysis to further explore whether the association between hypotension and POD might be modified by these factors.

## Results

**Figures 1:**
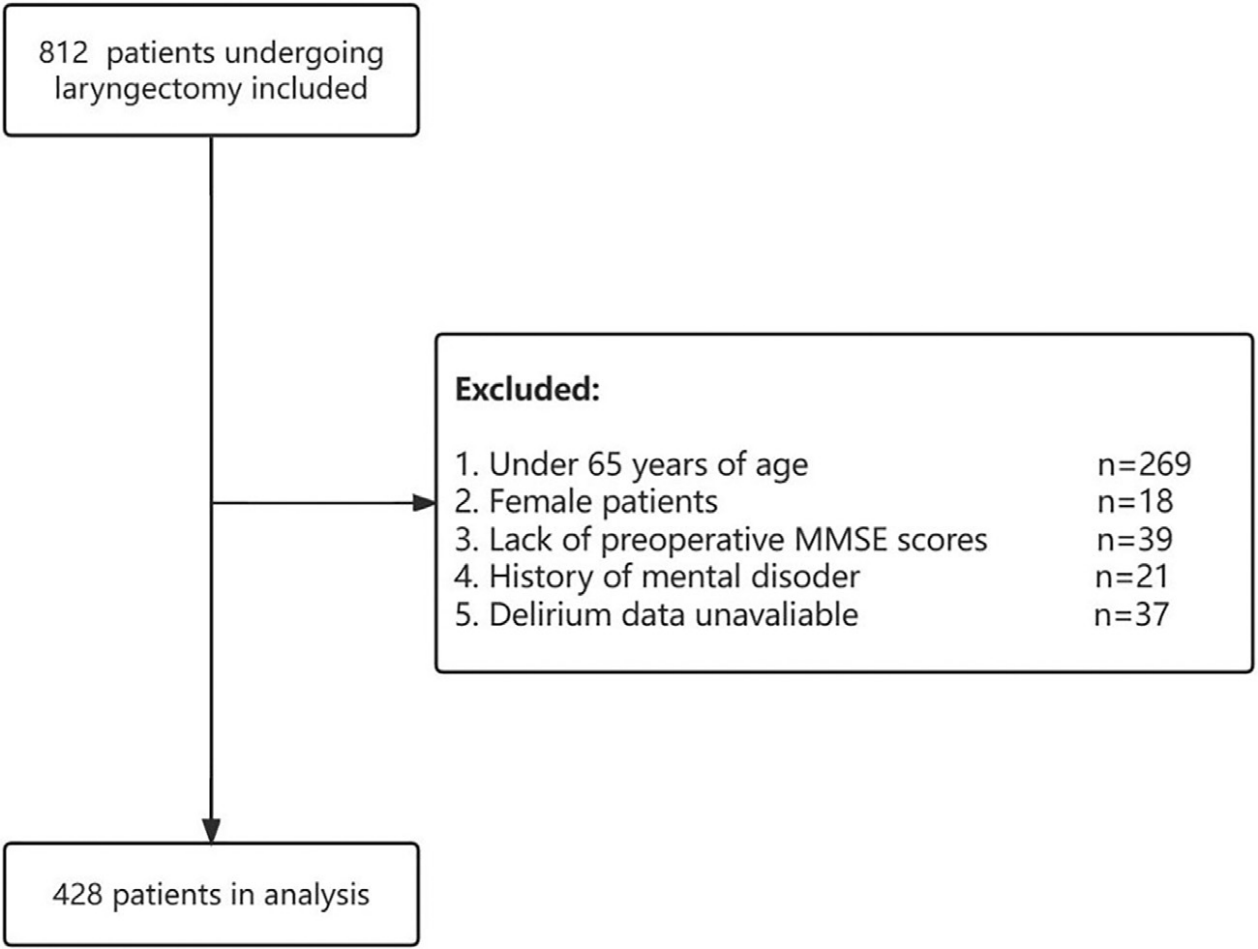
Diagram of trial recruitment with inclusion and exclusion criteria.

From April 2018 to January 2022, a total of 812 patients undergoing laryngectomy were included in this study. According to inclusion and exclusion criteria, data of 428 patients were finally analyzed (Fig. 1).

The demographic and clinical characteristics of the study population are summarized in Table 1. The mean age of the patients was 69.5 years (SD = 4.4 years). Among the patients, 39.5% had advanced-stage laryngeal cancer (stage III or IV), and 70.8% had low education levels. The mean MMSE score was 23.8 (SD = 6.2). The prevalence of comorbidities included 9.6% with a history of cerebral stroke, 44.6% with hypertension, 14.5% with diabetes mellitus, and 3.7% with a family history of dementia. The mean CIRs score was 5.4 (SD = 2.5). Additionally, 24.5% of patients reported poor sleep quality preoperatively, 88.3% of the patients were smokers, and 63.3% reported regular alcohol consumption. The median surgery duration was 209.6 minutes (IQR: 109–275 min), and 21.3% of the patients underwent surgery lasting 300 minutes or longer. Intraoperative dexmedetomidine was used in 49.3% of cases. A total of 11.7% of patients received more than 3000 mL of fluids intraoperatively, and 38.8% experienced hypotension during the procedure.

**Table 1:** Preoperative Variables Related to the Development of POD.

Factor	Total patients (n = 428)	POD (n = 77)	Non-POD (n = 351)	*p*-value^a^
Age	69.5 (4.4)	69.0 (3.8)	69.6 (4.5)	0.256
Cancer stage				0.293
I	117 (27.3%)	18 (23.4%)	99 (28.2%)	
II	142 (31.2%)	22 (28.6%)	120 (34.2%)	
III	106 (24.8%)	21 (27.3%)	85 (24.2%)	
IV	63 (14.7%)	16 (20.8%)	47 (13.4%)	
Education level				0.001
Low (less than high school)	303 (70.8%)	66 (85.7%)	237 (63.5%)	
High (greater than high school)	125 (29.2%)	11 (14.3%)	114 (32.5%)	
MMSE (< 27)	236 (55.1%)	52 (68.4%)	184 (52.6%)	0.012
CIRS (> 8)	77 (18.0%)	10 (13.0%)	67 (19.1%)	0.203
Previous cerebral stroke	41 (9.6%)	9 (11.7%)	32 (9.1%)	0.487
Hypertension	191 (44.6%)	27 (31.5%)	164 (46.7%)	0.062
Diabetes mellitus	62 (14.5%)	10 (13.0%)	52 (14.8%)	0.680
Smoke	378 (88.3%)	67 (87.0%)	311 (88.6%)	0.694
Drink	271 (63.3%)	47 (61.0%)	224 (64.0%)	0.625
Family history of dementia	16 (3.7%)	3 (3.9%)	13 (3.7%)	0.936
Preoperative poor sleep quality	105 (24.5%)	16 (20.8%)	89 (29.4%)	0.394
Surgery type				0.245
PL	134 (31.3%)	25 (32.5%)	109 (31.1%)	
PL and ND	100 (23.4%)	16 (20.8%)	84 (23.9%)	
TL	79 (18.5%)	9 (11.7%)	70 (19.9%)	
TL and ND	94 (21.9%)	21 (27.3%)	73 (20.4%)	
Total laryngopharyngectomy and ND	21(4.9%)	6 (7.8%)	15 (4.3%)	
Surgery duration ≥ 300 min	91 (21.3%)	24 (31.2%)	67 (19.1%)	0.019
Intraoperative hypotension	166 (38.8%)	38 (49.4%)	128 (36.5%)	0.036
Fluid infusion ≥ 3000 mL	50 (11.7%)	9 (11.7%)	41 (11.7%)	0.999
Intraoperative dexmedetomidine use	211 (49.3%)	36 (46.8%)	175 (51.0%)	0.5

Data are shown as mean (SD), or number (percentage).

MMSE, Mini-Mental State Examination; CIRS, Cumulative Illness Rating Scale; POD, Postoperative Delirium; PL, Partial Laryngectomy; ND, Neck Dissection; TL, Total Laryngectomy.

a Comparison between the POD group and the non-POD group.

The prevalence of POD was 18.0% (77/428) during the first five days postoperatively. Patients with POD were more likely to have low education levels (*p* = 0.001), lower MMSE scores (*p* = 0.012), prolonged surgery duration (*p* = 0.019), and intraoperative hypotension (*p* = 0.036; Table 1). The incidence of POD was not reduced by intraoperative dexmedetomidine use (*p* = 0.5).

**Figure 2:**
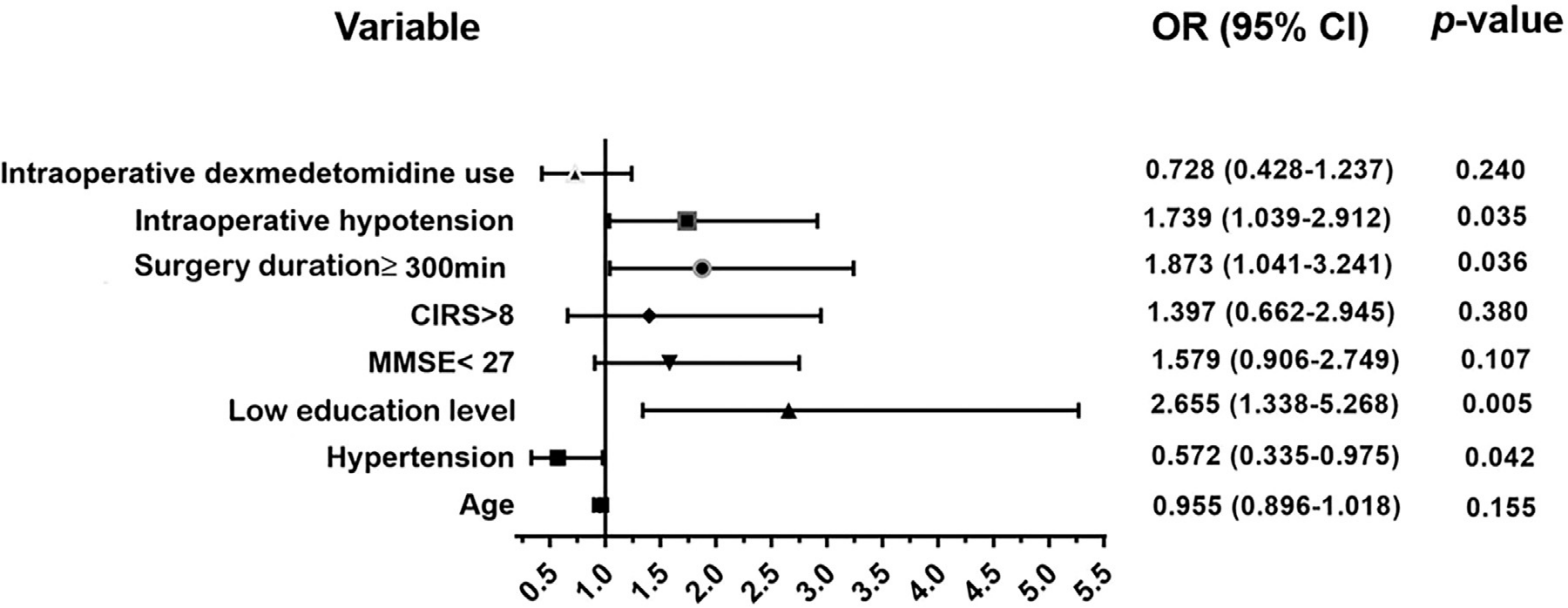
Forest plot of multivariate logistic regression analysis demonstrating factors associated with POD. POD, Postoperative Delirium.

Multivariate logistic regression model showed that low education level (OR = 2.655; 95% CI 1.338–5.268; *p* = 0.005), prolonged surgery duration (OR = 1.873; 95% CI 1.041–3.241; *p* = 0.036) and intraoperative hypotension (OR = 1.739; 95% CI 1.039–2.912; *p* = 0.035) were independently associated with occurrence of POD. However, preoperative hypertension appeared to be a protective factor for POD (OR = 0.572; 95% CI 0.335–0.975; *p* = 0.042) (Fig. 2).

The incidence of intraoperative hypotension in patients with or without POD was 49.4% (38 patients) and 36.5% (128 patients), respectively (*p* = 0.036, Table 1). The stratified analysis showed that the effect of intraoperative hypotension on POD varied in different strata of surgical duration (*p* for interaction = 0.008) rather than preoperative hypertension (*p* for interaction = 0.288) and education level (*p* for interaction = 0.198) (Table 2).

**Table 2:** Stratified analysis of the effect of intraoperative hypotension on POD.

Factors	Adjusted OR (95% CI)	*p* for interaction
Hypertension		0.288
No history of hypertension	1.424 (0.753‒2.698)	
History of hypertension	2.589 (1.116‒6.006)	
Education level		0.198
Low education level	1.421 (0.829‒2.462)	
High education level	3.642 (1.003‒13.224)	
Surgery duration		0.008
< 300 min	1.195 (0.660‒2.162)	
≥ 300 min	4.902 (1.816‒13.230)	

POD, Postoperative Delirium; OR, Odd Ratio.

## Discussion

Our study found that the prevalence of POD was 18.0% in elderly male patients after laryngectomy. Three risk factors were identified: intraoperative hypotension, low education level, and surgery duration of 300 minutes or longer. Intraoperative hypotension is clearly associated with an increase in the odds of POD and this association between intraoperative hypotension and POD was magnified in patients with prolonged surgery duration.

The adverse effect of intraoperative hypotension on the development of POD is not clear due to inconsistent definitions of hypotension and varying populations in previous studies.^31^, ^32^, ^33^ A retrospective study showed intraoperative hypotension (MAP ≤ 65 mmHg) for ≥ 5 min was associated with an increased incidence of POD after thoracic and orthopedic surgery in the elderly.^34^ In patients undergoing noncardiac surgery, a MAP < 55 mmHg was a risk factor for POD.^24^ Our previous observational study showed that intraoperative hypotension was a risk factor for POD in patients undergoing laryngectomy.^25^ In this study, we found that intraoperative hypotension increased the incidence of POD with an OR = 1.739 (95% CI 1.039–2.912). It is well recognized that the capability of cerebral autoregulation may be impaired in older patients.^35^^,^^36^ Hypotension results in reduction of cerebral perfusion pressure and mean cerebral blood flow velocity, and this effect is especially pronounced in elderly people with higher cardiovascular risk,^37^^,^^38^ which finally leads to cerebral dysfunction. Our finding supported that POD may be a manifestation of cerebral dysfunction due to temporary intraoperative cerebral under-perfusion^39^ and perioperative blood pressure should be strictly controlled in elderly patients.^40^

Previous studies determined that prolonged surgery duration was a risk factor for POD.^7^^,^^9^ Our analysis indicated that the association between intraoperative hypotension and POD was significantly influenced by surgery duration (*p* for interaction = 0.008). Specifically, the effect of intraoperative hypotension on the incidence of POD was more pronounced in patients who underwent prolonged surgeries (≥ 300 min). Consistent with a previous study,^24^ our finding suggests that patients who experience intraoperative hypotension during extended surgical procedures are at a higher risk of developing POD. Clinically, this underscores the importance of meticulous blood pressure management during long surgeries to mitigate the risk of POD. Prolonged surgeries may contribute to a cumulative stress response and increased vulnerability to hypotension-induced cerebral hypoperfusion, thereby elevating the risk of cognitive dysfunction postoperatively.

The underlying association between intraoperative hypotension and POD can be explained by several factors: reduced cerebral perfusion, especially in elderly patients with compromised cerebral autoregulation;^20^^,^^21^ the inflammatory response induced by surgical procedures;^37^ and pre-existing vulnerabilities such as cerebrovascular disease or other comorbidities.^8^^,^^12^

Education level and preoperative cognitive dysfunction have been identified as risk factors in POD studies.^25^^,^^41^^,^^42^ Higher education levels are associated with better cognitive reserve, potentially protecting against POD.^43^

A meta-analysis demonstrated a significant association between blood pressure reduction using antihypertensive agents and a reduced risk of incident dementia or cognitive impairment.^43^ However, the impact of preoperative hypertension on postoperative cognitive function is not yet fully understood. While some studies have suggested that preoperative hypertension was not associated with postoperative cognitive dysfunction,^44^^,^^45^ our findings indicated that preoperative hypertension may be a protective factor against POD (OR = 0.572, 95% CI 0.335–0.975). Specifically, we identified preoperative hypertension as a protective factor against POD, while intraoperative hypotension was found to be a risk factor. Chronic hypertension patients may experience reduced cerebral perfusion during intraoperative hypotension, potentially causing neuronal dysfunction. Moreover, these patients often have impaired cerebral autoregulation, making them more vulnerable to blood pressure fluctuations and thereby increasing the risk of POD. It is important to acknowledge the limitations of this retrospective study, such as potential selection bias and incomplete data, which may affect the generalizability of our findings. Further prospective studies are needed to validate these results and explore the underlying mechanisms in more detail.

Our study has several limitations. First, the data were exclusively from male patients, limiting generalizability. Second, POD incidence was recorded within five days postoperatively, without long-term cognitive assessment. Third, missing delirium data for 37 patients may affect robustness. Finally, intraoperative hypotension was recorded as a categorical variable without detailed analysis.

## Conclusion

In conclusion, our study demonstrates an association between intraoperative hypotension and POD in elderly male patients undergoing laryngectomy, particularly with prolonged surgery duration. Preventing intraoperative hypotension could be a modifiable risk factor for POD prevention in this patient population. Additionally, low educational level is also associated with POD. Future studies addressing the limitations mentioned, such as gender selection, time window for POD assessment, and detailed blood pressure analysis, are warranted to provide a more comprehensive understanding of the relationship between intraoperative hypotension and POD.

## Conflicts of interest

The authors declare no conflicts of interest.

## Appendix Supplementary materials

Image, application 1

Image, application 2

Image, application 3

## Footnotes

Supplementary material associated with this article can be found in the online version at doi:10.1016/j.bjane.2024.844560.
