# Supplementary material for: Intraoperative hypotension and postoperative delirium in elderly male patients undergoing laryngectomy: a single-center retrospective cohort study
Source: Braz J Anesthesiol. 2024 Sep 12;75(1):844560. doi: 10.1016/j.bjane.2024.844560 (PMC11440347; doi:10.1016/j.bjane.2024.844560)

**Mini-Mental State Examination (MMSE)**

**Instructions:** Score one point for each correct answer. Maximum score is 30 points.

1. **Orientation (10 points)**
   - **What is the year?** (1 point)
   - **What is the season?** (1 point)
   - **What is the date?** (1 point)
   - **What is the day of the week?** (1 point)
   - **What is the month?** (1 point)
   - **Where are we now (state)?** (1 point)
   - **What country are we in?** (1 point)
   - **What city/town are we in?** (1 point)
   - **What is the name of this place?** (1 point)
   - **What floor of the building are we on?** (1 point)
2. **Registration (3 points)**
   - **Name three objects: one second to say each. Then ask the patient all three after you have said them. Give one point for each correct answer. Repeat them until the patient learns all three. Count trials and record:**
     - **“Apple”** (1 point)
     - **“Penny”** (1 point)
     - **“Table”** (1 point)
3. **Attention and Calculation (5 points)**
   - **Serial 7s.** (1 point for each correct, stop after five answers): 93, 86, 79, 72, 65
   - **Alternatively:**
     - **Spell “WORLD” backwards.** (1 point for each correct letter): D, L, R, O, W
4. **Recall (3 points)**
   - **Ask for the three objects repeated above (Registration step). Give one point for each correct answer:**
     - **“Apple”** (1 point)
     - **“Penny”** (1 point)
     - **“Table”** (1 point)
5. **Language (9 points)**
   - Naming: Show a pencil and a watch

**and ask the patient to name them.** (2 points) - **Pencil** (1 point) - **Watch** (1 point)

- **Repeating: Ask the patient to repeat the following:**
  - **“No ifs, ands, or buts.”** (1 point)
- **Following a three-stage command:**
  - **“Take a paper in your right hand, fold it in half, and put it on the floor.”** (3 points)
    - **Take a paper in your right hand** (1 point)
    - **Fold it in half** (1 point)
    - **Put it on the floor** (1 point)
- **Reading and obeying:**
  - **“Please read this and do what it says.” (Show the patient the written instruction: “Close your eyes.”)** (1 point)
- **Writing:**
  - **“Please write a sentence.” (The sentence must contain a subject and a verb and make sense.)** (1 point)
- **Drawing:**
  - **“Please copy this drawing.” (Show the patient two overlapping pentagons.)** (1 point)


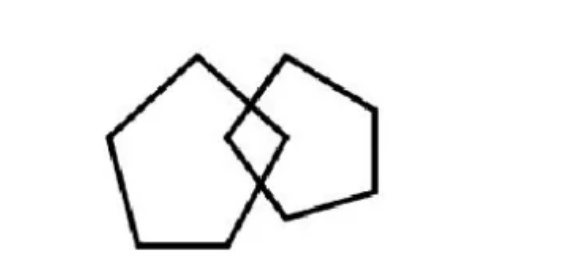

Supplement: Supplementary file 1 [file mmc1.docx]
