# Supplementary material for: Intraoperative hypotension and postoperative delirium in elderly male patients undergoing laryngectomy: a single-center retrospective cohort study
Source: Braz J Anesthesiol. 2024 Sep 12;75(1):844560. doi: 10.1016/j.bjane.2024.844560 (PMC11440347; doi:10.1016/j.bjane.2024.844560)
Supplement: Supplementary file 2 [file mmc2.docx]

**Cumulative Illness Rating Scale (CIRS)**

**Instructions:** Rate each of the following categories based on the severity of impairment. Scores range from 0 to 4, where:

- **0 = No problem affecting that system**
- **1 = Mild problem, does not interfere with normal activities**
- **2 = Moderate problem, interferes with normal activities**
- **3 = Severe problem, constant significant impact on normal activities**
- **4 = Extremely severe problem, immediate treatment required, or severe disability**

**Categories:**

1. **Heart (e.g., heart failure, angina)**
   - 0: No problem
   - 1: Mild problem
   - 2: Moderate problem
   - 3: Severe problem
   - 4: Extremely severe problem
2. **Vascular (e.g., hypertension, peripheral vascular disease)**
   - 0: No problem
   - 1: Mild problem
   - 2: Moderate problem
   - 3: Severe problem
   - 4: Extremely severe problem
3. **Hematopoietic (e.g., anemia, clotting disorders)**
   - 0: No problem
   - 1: Mild problem
   - 2: Moderate problem
   - 3: Severe problem
   - 4: Extremely severe problem
4. **Respiratory (e.g., asthma, chronic obstructive pulmonary disease)**
   - 0: No problem
   - 1: Mild problem
   - 2: Moderate problem
   - 3: Severe problem
   - 4: Extremely severe problem
5. **Eyes, Ears, Nose, Throat, and Larynx (e.g., vision or hearing loss)**
   - 0: No problem
   - 1: Mild problem
   - 2: Moderate problem
   - 3: Severe problem
   - 4: Extremely severe problem
6. **Upper Gastrointestinal (e.g., peptic ulcer disease, liver disease)**
   - 0: No problem
   - 1: Mild problem
   - 2: Moderate problem
   - 3: Severe problem
   - 4: Extremely severe problem
7. **Lower Gastrointestinal (e.g., irritable bowel syndrome, diverticulitis)**
   - 0: No problem
   - 1: Mild problem
   - 2: Moderate problem
   - 3: Severe problem
   - 4: Extremely severe problem
8. **Liver (e.g., cirrhosis, hepatitis)**
   - 0: No problem
   - 1: Mild problem
   - 2: Moderate problem
   - 3: Severe problem
   - 4: Extremely severe problem
9. **Kidney (e.g., chronic kidney disease, kidney stones)**
   - 0: No problem
   - 1: Mild problem
   - 2: Moderate problem
   - 3: Severe problem
   - 4: Extremely severe problem
10. **Genitourinary (e.g., prostate disease, urinary incontinence)**
    - 0: No problem
    - 1: Mild problem
    - 2: Moderate problem
    - 3: Severe problem
    - 4: Extremely severe problem
11. **Musculoskeletal/integumentary (e.g., arthritis, skin diseases)**
    - 0: No problem
    - 1: Mild problem
    - 2: Moderate problem
    - 3: Severe problem
    - 4: Extremely severe problem
12. **Neurological (e.g., stroke, epilepsy)**
    - 0: No problem
    - 1: Mild problem
    - 2: Moderate problem
    - 3: Severe problem
    - 4: Extremely severe problem
13. **Endocrine/metabolic (e.g., diabetes, thyroid disorders)**
    - 0: No problem
    - 1: Mild problem
    - 2: Moderate problem
    - 3: Severe problem
    - 4: Extremely severe problem
14. **Psychiatric (e.g., depression, anxiety)**
    - 0: No problem
    - 1: Mild problem
    - 2: Moderate problem
    - 3: Severe problem
    - 4: Extremely severe problem
15. **Other: Any other system not covered above (e.g., dental problems, infections)**
    - 0: No problem
    - 1: Mild problem
    - 2: Moderate problem
    - 3: Severe problem
    - 4: Extremely severe problem
