# Supplementary material for: Intraoperative hypotension and postoperative delirium in elderly male patients undergoing laryngectomy: a single-center retrospective cohort study
Source: Braz J Anesthesiol. 2024 Sep 12;75(1):844560. doi: 10.1016/j.bjane.2024.844560 (PMC11440347; doi:10.1016/j.bjane.2024.844560)
Supplement: Supplementary file 3 [file mmc3.docx]

**Confusion Assessment Method (CAM) Diagnostic Algorithm**

**Feature 1: Acute Onset and Fluctuating Course**

- **Is there evidence of an acute change in mental status from the patient's baseline?**
- **Did the abnormal behavior fluctuate during the day, that is, tend to come and go, or increase and decrease in severity?**

**Feature 2: Inattention**

- **Did the patient have difficulty focusing attention, for example, being easily distractible, or having difficulty keeping track of what was being said?**

**Feature 3: Disorganized Thinking**

- **Was the patient's thinking disorganized or incoherent, such as rambling or irrelevant conversation, unclear or illogical flow of ideas, or unpredictable switching from subject to subject?**

**Feature 4: Altered Level of Consciousness**

- **Is the patient’s level of consciousness anything other than alert, such as vigilant (hyperalert), lethargic (drowsy, easily aroused), stuporous (difficult to arouse), or comatose (unarousable)?**

**Scoring:**

To have a diagnosis of delirium by CAM, the patient must display:

- Features 1 and 2 **and**
- Either Feature 3 **or** Feature 4
